# Peripherally Inserted Central Catheters in Newborns: A Seven-Year Single-Center Experience from a Neonatal Intensive Care Unit

**DOI:** 10.3390/children12091168

**Published:** 2025-09-02

**Authors:** Hasan Avsar, Ali Bulbul, Evrim Kiray Bas, Hasan Sinan Uslu, Ebru Turkoglu Unal

**Affiliations:** 1 Department of Neonatology, Gaziantep Liv Hospital, 27080 Gaziantep, Turkey; 2 Department of Neonatology, Sisli Hamidiye Etfal Education and Research Hospital, University of Health Science, 34371 Istanbul, Turkey; ali.bulbul@sbu.edu.tr (A.B.); evrim.kiray.bas@sbu.edu.tr (E.K.B.); hasan.sinan.uslu@sbu.edu.tr (H.S.U.); ebru.turkoglu.unal@sbu.edu.tr (E.T.U.)

**Keywords:** neonate, peripherally inserted central catheter, ELBW, complications, CLABSI, catheter dwell time

## Abstract

**Highlights:**

**What are the main findings?**

• Extremely low birth weight (<1000 g), non-central catheter tip position, and temporal vein insertion are strong independent predictors of PICC-related complications in neonates.
• Use of antimicrobial-impregnated catheters significantly reduces complication risk and prolongs catheter dwell time compared to standard catheters.

**What is the implication of the main finding?**

• Targeted preventive strategies, optimal catheter tip positioning, and selective use of antimicrobial-impregnated catheters could improve PICC outcomes in NICU patients.
• High-risk groups, especially extremely low birth weight infants, require closer monitoring and strict adherence to insertion and maintenance bundles to reduce complications.

**Abstract:**

*Objectives*: To evaluate the clinical characteristics, complication rates, and predictors of adverse outcomes related to peripherally inserted central catheters (PICC) in newborns over a seven-year period in a tertiary neonatal intensive care unit (NICU). *Materials and Methods*: This retrospective observational study included all neonates who underwent their first PICC placement between January 2017 and January 2024 in a single tertiary NICU. Demographic, clinical, and procedural data were collected, including birth weight, gestational age, catheter type, insertion site, dwell time, and reason for removal. Primary outcomes were PICC-related complications and catheter dwell time. Statistical analyses included chi-square or Fisher’s exact tests, Student’s t-test or ANOVA, and multivariable logistic regression to identify independent predictors of complications. *Results*: A total of 610 PICCs were evaluated. The mean gestational age was 31.0 ± 4.7 weeks, and the mean birth weight was 1579 ± 870 g. The majority of catheters (96.1%) terminated at the central location, with a mean dwell time of 12.9 ± 9.0 days. The most common removal reason was completion of therapy (60.3%), followed by mechanical complications (36.7%) and suspected infection (14.6%). Overall complication rate was 34.9%. In multivariable analysis, birth weight > 1000 g was associated with a lower risk of complications compared to <1000 g (1000–1500 g: OR 0.35, 95% CI 0.22–0.56; 1500–2000 g: OR 0.29, 0.15–0.54; >2000 g: OR 0.44, 0.21–0.92). Midline (OR 4.16, 1.76–9.83) and peripheral (OR 3.51, 1.82–6.76) terminations carried higher risk compared to central terminations. Use of antimicrobial-impregnated catheters reduced complication risk (OR 0.44, 0.26–0.74), while temporal vein insertion increased it (OR 4.14, 1.23–13.86). *Conclusions*: Low birth weight (<1000 g) and non-central catheter tip location are significant predictors of PICC-related complications in neonates, while antimicrobial-impregnated catheters have a protective effect. Targeted preventive strategies, strict adherence to insertion and maintenance bundles, and careful patient selection are recommended to improve outcomes in NICU patients.

## 1. Introduction

Peripherally inserted central catheters (PICC) have become an essential component of neonatal intensive care unit (NICU) management, particularly for premature and critically ill newborns requiring prolonged intravenous therapy, parenteral nutrition, or administration of irritant medications. Compared to other central venous access methods, PICCs offer the advantages of bedside insertion, lower risk of major mechanical complications, and extended dwell time, while avoiding repeated peripheral cannulations that can cause significant discomfort and tissue injury in neonates [1,2,3,4,5].

Since their introduction into neonatal care in the late 1970s, PICCs have been increasingly preferred for very low birth weight (VLBW) and extremely low birth weight (ELBW) infants, as these populations often require long-term vascular access for nutritional and therapeutic support [6,7,8]. Multiple studies have demonstrated that PICCs are associated with reduced procedural stress, improved maintenance of intravenous therapy, and, when inserted and managed with strict aseptic techniques, acceptable rates of complications such as catheter-related bloodstream infections (CRBSI), thrombosis, and mechanical dysfunction [9,10,11,12,13].

However, despite their advantages, PICCs are not without risks. Reported complication rates vary between 10% and 30% depending on patient population, insertion technique, and maintenance protocols [14]. Infectious complications remain a significant concern, as neonates—particularly those with prematurity—have immature immune systems and prolonged NICU stays, both of which increase vulnerability to CRBSI [15,16]. Non-infectious complications, including occlusion, dislodgement, and phlebitis, can also lead to premature catheter removal and treatment interruption [17].

The decision to use a PICC is influenced by multiple factors, including gestational age, birth weight, primary diagnosis, and the type of therapy planned. Moreover, the site of insertion, catheter size, and type are known to affect catheter dwell time and complication rates [18,19]. While previous single-center studies have described PICC use in neonates, most have been based on relatively small patient cohorts and limited follow-up periods [20,21,22].

In 2010, our institution published its initial experience with PICC use in the NICU, based on a smaller dataset and shorter follow-up [23]. Since then, our practice has evolved with increased clinical experience, updated insertion protocols, and the adoption of preventive strategies aimed at reducing catheter-related complications. The present study aimed to provide an updated and comprehensive analysis of our 7-year experience with PICC use in newborns, including data from PICC insertions, evaluating catheter dwell time, complication rates, and the impact of patient and catheter characteristics on outcomes.

## 2. Materials and Methods

### 2.1. Study Design and Patient Selection

This retrospective observational study was conducted in the neonatal intensive care unit (NICU) of Şişli Hamidiye Etfal Training and Research Hospital, a tertiary referral center, over a 7-year period from 1 January 2017 to 1 January 2024. The NICU provides care for preterm and term newborns requiring intensive medical and surgical support, including advanced respiratory care, parenteral nutrition, and invasive monitoring.

All neonates admitted to the NICU during the study period who received a PICC were eligible for inclusion. Patients were identified from the unit’s catheter insertion registry and cross-referenced with the electronic medical record system. Only the first PICC insertion episode for each patient was included in the analysis to avoid duplication. Newborns who received other types of central venous catheters (e.g., surgically placed tunneled catheters, umbilical venous catheters) or whose records were incomplete were excluded. A total of 610 PICC insertions meeting the inclusion criteria were analyzed.

Data were collected retrospectively from patient charts and NICU catheter procedure records using a standardized data extraction form. Recorded variables included demographic information such as gestational age, birth weight, sex, and mode of delivery; clinical data including primary diagnosis at admission, comorbidities, indication for PICC insertion, and the need for other vascular access (e.g., umbilical catheters); and catheter characteristics such as insertion date, catheter type (standard or antibiotic-impregnated), size (1 Fr or 2 Fr), insertion site and vein used, number of insertion attempts, side of insertion, dwell time, and reason for removal. Complications were defined as any infection, thrombosis, occlusion, mechanical dislodgement, or other catheter-related event resulting in premature removal. Infectious complications were further classified as CRBSI, clinical sepsis without laboratory confirmation, or positive catheter tip cultures, and all organisms isolated from blood or catheter cultures were documented.

### 2.2. PICC Insertion Protocol

All PICC insertions were performed at the bedside in the NICU by trained neonatal staff, including neonatologists or specialized neonatal nurses, in accordance with the unit’s aseptic insertion protocol. Preparation involved maximal barrier precautions, including the use of a sterile gown, gloves, mask, and cap, with the infant draped in a sterile fashion. Skin antisepsis was performed with chlorhexidine gluconate or povidone-iodine, depending on the infant’s age and weight. Catheter size and type were selected based on the infant’s weight and therapeutic requirements. For insertion, the basilic, cephalic, or another accessible peripheral vein in the upper or lower limb was cannulated, using ultrasound guidance when available, and the catheter tip position was confirmed by anteroposterior chest radiograph. Maintenance included daily inspection of the insertion site and dressing integrity, routine flushing with heparinized saline, and replacement of administration sets every 72 h in accordance with CDC guidelines [24]. Removal was indicated upon completion of therapy, occurrence of complications, or patient death.

### 2.3. Definitions

**Figure 1:**
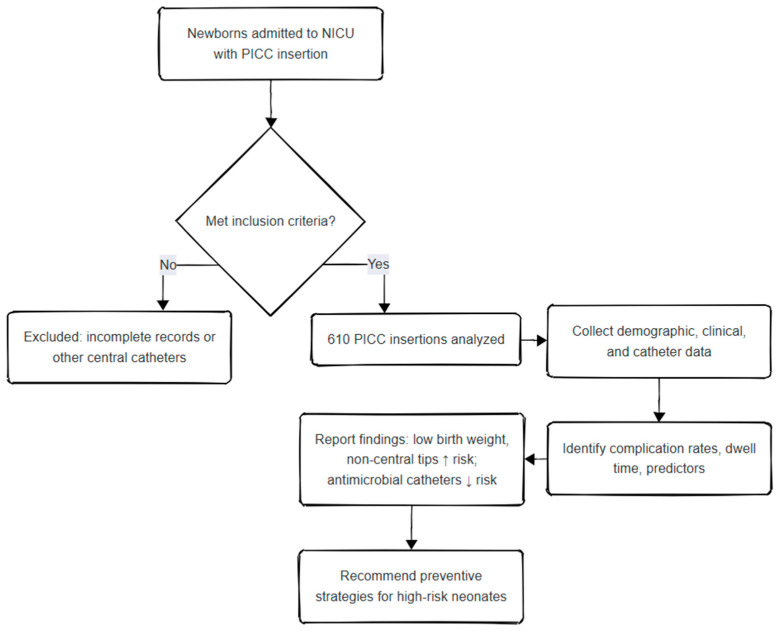
Flowchart of the Study Design.

Catheter dwell time was defined as the number of days from PICC insertion to removal. The complication rate was calculated as the number of PICC insertions with any complication divided by the total number of insertions in the group. Catheter-related bloodstream infection (CRBSI) was defined according to CDC criteria as a laboratory-confirmed bloodstream infection in a patient with a central line in place at the time of, or within 48 h before, the onset of infection, with no other identifiable source. Catheter-associated bloodstream infection (CLABSI) refers to any bloodstream infection occurring while a PICC was in place, regardless of strict causality [24]. Infectious complications were further classified as CRBSI, clinical sepsis without laboratory confirmation, or positive catheter tip cultures, and all organisms isolated from blood or catheter cultures were documented (Figure 1).

### 2.4. Statistical Analysis

All statistical analyses were performed using IBM SPSS Statistics for Windows, Version 27.0 (IBM Corp., Armonk, NY, USA). Continuous variables were expressed as mean ± standard deviation (SD) or median (interquartile range, IQR) depending on distribution. Categorical variables were presented as frequencies and percentages. Differences in complication rates between groups were analyzed using the chi-square test or Fisher’s exact test when appropriate. Differences in catheter dwell time between two groups were analyzed using the independent samples t-test, and between more than two groups using one-way analysis of variance (ANOVA). Correlation analyses between duration of catheter life and clinical variables (birth weight, insertion site, catheter type, and primary diagnosis) were performed using Spearman’s rank correlation coefficient for non-parametric data. Univariate logistic regression analyses were performed to identify potential predictors of catheter-related complications. Variables with *p* < 0.10 in univariate analysis were entered into a multivariate logistic regression model to identify independent risk factors. Odds ratios (OR) with 95% confidence intervals (CI) were calculated. A two-tailed *p*-value < 0.05 was considered statistically significant.

## 3. Results

Based on the demographic characteristics of the 610 newborns who underwent PICC insertion, the mean gestational age was 31.0 ± 4.7 weeks (range: 22–41), with a mean birth weight of 1579.2 ± 869.6 g (range: 300–4000 g). Male infants accounted for 54.6% (*n* = 333) of the cohort. Cesarean section was the predominant mode of delivery (68.0%), followed by normal spontaneous delivery (32.0%). Very low birth weight infants (<1000 g) constituted 33.0% of the study population, while 29.5% weighed >2000 g at birth. Prematurity was the most frequent primary diagnosis (75.6%), followed by necrotizing enterocolitis (15.7%) and sepsis (3.9%). The leading indication for PICC insertion was total parenteral nutrition (62.1%), with antibiotic therapy alone in 23.9% and combined therapy in 13.9% of cases. The mean body weight at the time of catheter insertion was 1663.7 ± 898.7 g (range: 400–6145 g). PICCs were placed at a mean of 8.3 ± 11.3 days of life (range: 0–82) and remained in situ for a mean of 12.9 ± 9.0 days (range: 0–100). The mean duration of parenteral nutrition was 13.0 ± 9.0 days, and the mean number of antibiotic courses administered was 15.4 ± 14.2 (range: 0–113) (Table 1).

In the analysis of PICC insertion characteristics, the majority of catheters used were standard (82.3%), with antibiotic-impregnated catheters accounting for 17.7% of cases. The 1 French size catheter was overwhelmingly preferred (92.3%), while 2 French size was used in only 7.7% of insertions. The right side was the predominant site of insertion (70.5%), compared to the left side (29.5%). The basilic vein was the most frequently cannulated vessel (45.6%), followed by the cephalic vein (24.1%) and axillary vein (16.6%). Less common sites included the jugular vein (7.0%), temporal vein (3.3%), femoral vein (2.0%), saphenous vein (1.0%), and subclavian vein (0.5%). Almost all catheters (96.1%) were positioned centrally, with midline (2.8%) and peripheral (1.1%) placements being rare. Most PICCs were successfully inserted on the first attempt (86.1%), with 10.0% requiring two attempts, 2.5% three attempts, and 1.5% more than three attempts (Table 2).

Reasons for removal of PICC and PICC related complications were shown in Table 3. The most frequent reason for PICC removal was that the catheter was no longer needed (elective removal), accounting for 60.3% (*n* = 368) of cases. Mechanical complications represented the second most common reason (36.7%, *n* = 224), with local phlebitis (*n* = 86) and occlusion of the catheter (*n* = 43) being the leading subtypes, followed by leakage or breakage (*n* = 23), complete distal dislodgement (*n* = 14), and catheter migration (*n* = 4). Suspected infection was reported in 14.6% (*n* = 89) of cases, all classified as catheter-related bloodstream infections (BSI). Additionally, 156 cases of catheter-associated BSI were recorded during the study period. Suspected thrombosis was rare, observed in only five cases (0.8%) (Table 3).

Comparison of complication rates and catheter life duration according to patient and catheter characteristics were shown in Table 4. In the comparative analysis of complication rates and catheter survival times across different patient and catheter characteristics, birth weight showed a significant impact on both outcomes. Infants weighing <1000 g exhibited the highest complication rate (50.7%) and the longest mean catheter duration (16.38 ± 11.60 days), whereas those weighing > 2000 g had shorter catheter life (10.97 ± 7.50 days) and lower complication rates (33.3%) (*p* < 0.001 for both). Catheter size did not significantly affect complication rates or duration (*p* = 0.774 and *p* = 0.165, respectively). Insertion site was a significant factor, with peripheral insertions showing the highest complication rate (60.7%) and the shortest duration (9.57 ± 6.94 days), while central insertions had lower complication rates (32.6%) and longer duration (13.86 ± 9.69 days) (*p* < 0.001). Catheter type also influenced outcomes; antibiotic-impregnated catheters had lower complication rates (26.9%) and longer duration (15.98 ± 9.58 days) compared to standard catheters (*p* = 0.021 for complication rate, *p* = 0.001 for duration). Delivery type did not significantly affect outcomes. Among primary diagnoses, prematurity was the most common and showed moderate complication rates (40.0%) with a mean catheter duration of 14.26 ± 10.11 days. Vein choice significantly influenced complication rates (*p* = 0.018), with temporal vein use associated with the highest complication rate (75.0%) and basilic/cephalic veins demonstrating more favorable outcomes (Table 4).

**Figure 2:**
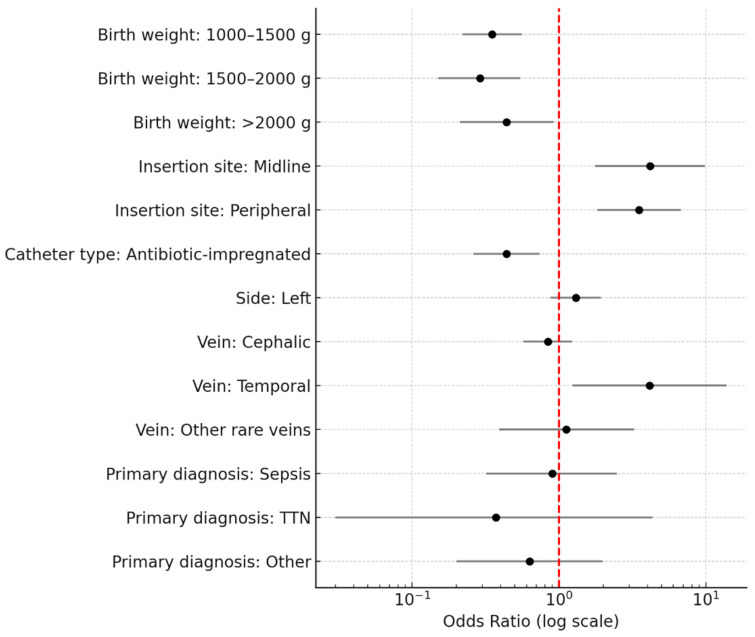
Factors Associated with PICC Complications.

In the multivariable logistic regression analysis (Table 5), lower birth weight was significantly associated with a higher risk of PICC-related complications. Compared with infants weighing < 1000 g, those with a birth weight of 1000–1500 g, 1500–2000 g, and >2000 g had significantly lower odds of complications (OR = 0.35, 0.29, and 0.44, respectively; all *p* < 0.05). Insertion site was another strong predictor: midline and peripheral insertions were associated with a 4.16-fold and 3.51-fold higher risk of complications, respectively, compared to central insertions (*p* ≤ 0.001). Use of antibiotic-impregnated catheters was protective, reducing the odds of complications by 56% (OR = 0.44, *p* = 0.002). Regarding the venous access site, temporal vein use was associated with a markedly higher complication risk (OR = 4.14, *p* = 0.021) compared with basilic vein access. Side of insertion (left vs. right), other vein types, and primary diagnosis (sepsis, transient tachypnea of the newborn, or other diagnoses vs. prematurity) were not independently associated with complications after adjustment (Table 5, Figure 2).

## 4. Discussion

In this large, single-center retrospective study spanning seven years and including 610 neonates with peripherally inserted central catheters (PICC), we identified several factors independently associated with catheter-related complications. Extremely low birth weight (<1000 g), non-central tip positions, and use of standard (non-antibiotic) catheters significantly increased complication risk, while antibiotic-impregnated catheters were protective. The mean time to first PICC insertion in our cohort was 8.3 days, which is relatively later than in some other reports. This delay can be attributed to our practice of initially using umbilical venous or peripheral intravenous access, with PICCs reserved for infants requiring prolonged therapy. In addition, PICC placement was occasionally postponed until clinical stabilization, which also contributed to the later timing.

Pet et al. reported that lower birth weight is a major risk factor for PICC-related complications in neonates, likely due to small vessel caliber, immature skin barrier, and increased fragility [25]. Shalabi et al. found higher rates of both infectious and mechanical complications in extremely low birth weight (ELBW) infants [20]. Our findings corroborate these observations, as infants under 1000 g had the highest complication rates. In contrast, Masuda et al. did not observe a statistically significant association between birth weight and catheter survival, which they attributed to strict insertion and maintenance protocols in their NICU [26].

Cartwright et al. demonstrated that central venous placement via basilic or cephalic veins was associated with longer catheter dwell times and lower complication rates compared to peripheral or malpositioned tips [21]. Chopra et al. highlighted the importance of optimal tip position for preventing PICC-associated bloodstream infections [14]. In our cohort, midline and peripheral placements increased the odds of complications by more than threefold, supporting these earlier findings. Kim et al. and Chhina et al. also emphasized that inappropriate tip position predisposes to mechanical issues and thrombosis, reinforcing our results [9,10].

Lai et al. reported that antibiotic-impregnated catheters reduced the risk of CRBSI in neonates, consistent with our finding of a 56% lower complication rate in this group [27]. Catho et al. and Chopra et al. also documented device-related differences in infection risk [12,14]. However, Loveday et al. and Timsit et al. caution that antimicrobial devices should be reserved for high-risk cases due to cost and antimicrobial stewardship considerations [5,28]. Although our findings demonstrate a protective effect of antimicrobial-impregnated catheters, the retrospective nature of this study precludes causal inference. Beyond clinical outcomes, the use of antimicrobial-impregnated catheters also raises economic and ethical considerations. These devices incur higher direct costs compared with standard catheters, and their routine implementation may not be feasible in all healthcare systems, particularly in resource-limited settings. From an ethical perspective, their use must be justified not only by reduced complication rates but also by demonstrable cost-effectiveness, ensuring equitable access to neonatal intensive care resources. In addition, concerns about antimicrobial stewardship and the potential for resistance development underscore the need for judicious use. Therefore, antimicrobial catheters should be reserved for high-risk populations where the benefits clearly outweigh the risks, and well-designed randomized controlled trials are required to confirm both their clinical efficacy and long-term cost-effectiveness in neonatal intensive care settings.

Rosenthal et al., Liu et al., and Hawes et al. emphasized that adherence to evidence-based insertion and maintenance bundles—including maximal barrier precautions, daily site inspection, and timely dressing changes—is crucial for reducing catheter-related complications [29,30,31]. Zang et al. found that guideline compliance during insertion and maintenance was directly associated with lower CLABSI rates [3]. Our relatively low complication rate compared to the earlier institutional data [23] may reflect improvements in bundle adherence, regular heparinized saline flushing, and replacement of administration sets every 72 h, in line with CDC recommendations [32,33,34]. No cases of heparin-induced thrombocytopenia (HIT) were observed in our cohort. Although the incidence of HIT is reported to be very low in neonates due to immune system immaturity, its potential risk has led some centers to abandon routine heparinization protocols. This issue should be considered when evaluating catheter maintenance strategies in neonatal intensive care units.

Ainsworth et al. reported a mean catheter dwell time of 12.3 days with a complication rate of 35%, values closely matching our findings (12.9 days; 34.9% complications) [17]. Chopra et al. and Badheka et al. also documented similar rates in pediatric and neonatal populations, suggesting our outcomes are within an expected range for high-acuity NICUs [13,14].

The collective evidence supports prioritizing central venous access with proper tip positioning, heightened surveillance for ELBW infants, and considering antibiotic-impregnated catheters in high-risk cases. Early recognition and management of complications remain essential, as supported by Vasudevan et al. advocated early removal in confirmed CRBSI when clinically feasible [8].

Compared with previous reports, the present study provides a larger, more recent, and more comprehensive dataset on PICC use in neonates, spanning a seven-year period and including 610 first-time PICC insertions in a single tertiary NICU. While earlier studies such as Pet et al. and Shalabi et al. identified low birth weight as a risk factor [20,25], our study not only confirmed this association but also quantified the impact across multiple birth weight subgroups using multivariable analysis. In addition, we demonstrated that both midline and peripheral tip positions significantly increased complication risk—findings that build upon the observations of Cartwright et al. and Chopra et al. by providing specific odds ratios for each non-central location [14,21]. Another distinguishing aspect of our work is the evaluation of antibiotic-impregnated catheters in this population. While Lai et al. reported their effectiveness in reducing CRBSI, few studies have quantified their protective effect alongside other clinical and procedural variables in a multivariate model; our analysis showed a 56% risk reduction [27]. Furthermore, unlike earlier studies with limited data on rare insertion sites, our dataset includes outcomes for less common veins such as the temporal vein, allowing us to identify it as an independent risk factor (OR 4.14). Our data revealed that temporal vein insertions carried a markedly higher complication risk than basilic or cephalic vein access. Although temporal vein cannulation is occasionally used when other veins are inaccessible, our findings suggest that it should be considered a high-risk site, and its use should be limited to selected cases when safer alternatives are unavailable. Finally, the relatively low overall complication rate in our cohort compared to historical institutional data suggests that adherence to updated insertion and maintenance bundles may have contributed to improved outcomes—highlighting a temporal improvement not fully explored in the previous literature.

### Limitations of the Study

This study has some limitations. First, its retrospective, single-center design may limit the generalizability of the findings to other neonatal intensive care units with different patient populations, insertion protocols, or maintenance practices. Second, although we included a large sample size, certain subgroups—such as rare insertion sites (temporal or subclavian veins) and unusual primary diagnoses—had small numbers, which may have limited the statistical power to detect significant associations. Third, we did not capture detailed data on operator experience or compliance with insertion and management bundles, both of which are potential confounders. Finally, differences in local protocols, personnel structures, and healthcare systems across facilities and countries may affect catheter-related outcomes, and these contextual factors should be considered when translating our findings into clinical practice. Finally, differences in local protocols, personnel structures, and healthcare systems across facilities and countries may affect catheter-related outcomes, and these contextual factors should be considered when translating our findings into clinical practice. Strengths include the large sample size, long study period, and multivariate modeling to adjust for confounders.

## 5. Conclusions

In conclusion, peripherally inserted central catheters are an essential component of neonatal intensive care, yet they remain associated with a considerable complication burden, particularly in extremely low birth weight infants and in cases where the catheter tip is not positioned centrally. Our findings highlight that the use of antimicrobial-impregnated catheters confers a protective effect, while temporal vein insertion is associated with increased risk. Targeted preventive strategies, meticulous insertion techniques, and strict adherence to maintenance bundles are crucial to improving PICC outcomes in this vulnerable population. Future multicenter, prospective studies, including randomized controlled trials, are warranted to validate these findings and to develop evidence-based guidelines tailored to high-risk neonates.

## Figures and Tables

**Table 1:** The demographic characteristics of the study group (*n* = 610).

Variable	Mean ± SD or *n* (%)	Range
Gender		
Male	333 (54.6)	
Female	277 (45.4)	
Gestational age (weeks)	31.0 ± 4.7	22–41
Delivery type		
Cesarean Section (C/S)	415 (68.0)	
Normal Spontaneous Delivery (NSD)	195 (32.0)	
Birth weight (g)	1579.2 ± 869.6	300–4000
Birth weight subgroups		
<1000 g	201 (33.0)	
>2000 g	180 (29.5)	
1000–1500 g	155 (25.4)	
1500–2000 g	74 (12.1)	
Primary diagnosis		
Prematurity	461 (75.6)	
Necrotizing Enterocolitis (NEC)	96 (15.7)	
Other	24 (3.9)	
Sepsis	24 (3.9)	
Transient Tachypnea of the Newborn (TTN)	4 (0.7)	
Feeding intolerance	1 (0.2)	
Catheter indication		
Total Parenteral Nutrition (TPN)	379 (62.1)	
Antibiotic therapy	146 (23.9)	
TPN + Antibiotic	85 (13.9)	
Body weight at the catheter insertion (g)	1663.7 ± 898.7	400–6145
Day of life PICC placed (days)	8.3 ± 11.3	0–82
Duration of PICC (days)	12.9 ± 9.0	0–100
Duration of PN (days)	13.0 ± 9.0	0–100
Number of applied antibiotic (times)	15.4 ± 14.2	0–113

**Table 2:** Catheter Insertion Characteristics (PICC Patients).

Variable	*n* (%)
Catheter type	
Standard	502 (82.3)
Antibiotic	108 (17.7)
Catheter size	
1 French	563 (92.3)
2 French	47 (7.7)
Side	
Right	430 (70.5)
Left	180 (29.5)
Vein	
Basilic vein	278 (45.6)
Cephalic vein	147 (24.1)
Axillary vein	101 (16.6)
Jugular vein	43 (7.0)
Temporal vein	20 (3.3)
Femoral vein	12 (2.0)
Saphenous vein	6 (1.0)
Subclavian vein	3 (0.5)
Catheter site	
Central	586 (96.1)
Midline	17 (2.8)
Peripheral	7 (1.1)
Number of attempts	
1 attempt	525 (86.1)
2 attempts	61 (10.0)
3 attempts	15 (2.5)
>3 attempts	9 (1.5)

**Table 3:** Reasons for removal of PICC and PICC related complications.

Reasons for Removal of PICC	Frequency, *n* (%)
No longer needed (Elective)	368 (60.3)
Mechanical complications	224 (36.7)
Occlusion of the catheter	43
Leakage or breakage	23
Catheter migration	4
Local phlebitis	86
Complete distal dislodgement	14
Suspected infection *	89 (14.6)
Catheter-related BSI ^†^	89 (14.6)
Catheter associated BSI ^‡^	156 (25.6)
Suspected thrombosis	5
Total	610 (100)

* Suspected infection: refers to PICC removals due to clinical suspicion of infection (*n* = 89, 14.6%). † Catheter-related BSI (CRBSI): all 89 suspected infections were microbiologically confirmed as CRBSI, defined according to CDC criteria. ‡ Catheter-associated BSI: total number of bloodstream infections documented during the study period while a PICC was in place (*n* = 156), including cases not directly resulting in catheter removal.

**Table 4:** Comparison of complication rates and catheter life duration according to patient and catheter characteristics in 610 PICC insertions.

Variables	Number of Catheters, *n* (%)	Complication Rate, *n* (%)	Duration of Catheter Life, Day (Mean ± SD)
**Birth weight**			
<1000 g	201 (33.0)	102 (50.7)	16.38 ± 11.60
1000–1500 g	154 (25.3)	41 (26.6)	13.04 ± 8.68
1500–2000 g	74 (12.1)	23 (31.1)	11.23 ± 5.89
>2000 g	180 (29.6)	60 (33.3)	10.97 ± 7.50
***p*** **value**		<0.001 *	<0.001 ʈ
**Catheter size**			
1 French	563 (54.2)	210 (37.3)	13.4 ± 9.7
2 French	47 (4.5)	16 (34.0)	12.0 ± 6.3
***p*** **value**		0.774 *	0.165 ʈ
**Insertion site**			
Central	521 (85.6)	170 (32.6)	13.86 ± 9.69
Midline	32 (5.3)	22 (68.8)	10.97 ± 7.62
Peripheral	56 (9.2)	34 (60.7)	9.57 ± 6.94
***p*** **value**		<0.001 *	0.001 ʈ
**Catheter type**			
Standard	501 (82.3)	197 (39.3)	12.73 ± 9.34
Antibiotic	108 (17.7)	29 (26.9)	15.98 ± 9.58
***p*** **value**		0.021 *	0.001 ʈ
**Delivery type**			
Cesarean Section (C/S)	415 (68.1)	143 (34.5)	13.49 ± 9.65
Normal Spontaneous Delivery (NSD)	194 (31.9)	83 (42.8)	12.92 ± 9.05
***p*** **value**		0.065 *	0.481 ʈ
**Primary diagnosis**			
Prematurity	460 (76.4)	184 (40.0)	14.26 ± 10.11
Sepsis	24 (3.9)	8 (33.3)	11.58 ± 7.83
TTN	4 (0.7)	1 (25.0)	8.00 ± 2.12
NEC	15 (2.5)	4 (26.7)	8.00 ± 3.48
***p*** **value**		0.086 *	0.005 ʈ
**Vein**			
Basilic vein	248 (40.7)	94 (37.9)	12.65 ± 8.38
Cephalic vein	327 (53.7)	111 (33.9)	13.68 ± 9.86
Axillary vein	4 (0.7)	2 (50.0)	25.50 ± 24.75
Jugular vein	4 (0.7)	2 (50.0)	14.25 ± 5.40
Temporal vein	20 (3.3)	15 (75.0)	12.05 ± 9.08
***p*** **value**		0.018 *	0.217 ʈ

Complication rate: percentage calculated as number of catheters with complications divided by total catheters in each subgroup. BSI data: include both catheter-related BSI (CRBSI, microbiologically confirmed) and catheter-associated BSI (all bloodstream infections during PICC presence). *p* *: Chi-square test (Complication rate); *p* ʈ: *t*-test (2 group)/ANOVA (3+ group).

**Table 5:** Logistic regression: factors associated with PICC complications.

Variable (Reference)	Category (Comparison)	OR	95% CI	*p*-Value
Birth weight (<1000 g)	1000–1500 g	0.35	0.22–0.56	<0.001
	1500–2000 g	0.29	0.15–0.54	<0.001
	>2000 g	0.44	0.21–0.92	0.028
Insertion site (Central)	Midline	4.16	1.76–9.83	0.001
	Peripheral	3.51	1.82–6.76	<0.001
Catheter type (Standard)	Antibiotic-impregnated	0.44	0.26–0.74	0.002
Side (Right)	Left	1.30	0.87–1.93	0.200
Vein (Basilic)	Cephalic	0.84	0.57–1.22	0.355
	Temporal	4.14	1.23–13.86	0.021
	Other (pooled rare veins)	1.12	0.39–3.24	0.832
Primary diagnosis (Prematurity)	Sepsis	0.90	0.32–2.48	0.835
	TTN	0.37	0.03–4.33	0.426
	Other	0.63	0.20–1.98	0.434

## Data Availability

The datasets generated and analyzed during the current study are not publicly available due to patient privacy and ethical restrictions but are available from the corresponding author on reasonable request.
